# Protein–peptide complex crystallization: a case study on the ERK2 mitogen-activated protein kinase

**DOI:** 10.1107/S0907444912051062

**Published:** 2013-02-16

**Authors:** Gergő Gógl, Imre Törő, Attila Reményi

**Affiliations:** a Department of Biochemistry, Eötvös Loránd University, Pázmány Péter sétány 1/C, 1117 Budapest, Hungary

**Keywords:** linear motifs, surface engineering, ERK2, protein–peptide complexes

## Abstract

Linear motifs normally bind with only medium binding affinity (*K*_d_ of ∼0.1–10 µ*M*) to shallow protein-interaction surfaces on their binding partners. The crystallization of proteins in complex with linear motif-containing peptides is often challenging because the energy gained upon crystal packing between symmetry mates in the crystal may be on a par with the binding energy of the protein–peptide complex. Furthermore, for extracellular signal-regulated kinase 2 (ERK2) the protein–peptide docking surface is comprised of a small hydrophobic surface patch that is often engaged in the crystal packing of apo ERK2 crystals. Here, a rational surface-engineering approach is presented that involves mutating protein surface residues that are distant from the peptide-binding ERK2 docking groove to alanines. These ERK2 surface mutations decrease the chance of ‘unwanted’ crystal packing of ERK2 and the approach led to the structure determination of ERK2 in complex with new docking peptides. These findings highlight the importance of negative selection in crystal engineering for weakly binding protein–peptide complexes.

## Abstract 2

A rational surface-engineering approach led to the crystal structure determination of ERK2–docking peptide complexes.

## 1. Introduction

Linear motifs (LMs) are simple protein–protein interaction tools which are generally less than 20 amino acids in length. They normally bind with only medium binding affinity (*K*_d_ of ∼0.1–10 µ*M*) to shallow protein-interaction surfaces on their binding partners (Neduva & Russell, 2005 ▶). As these protein–peptide-type inter­actions are becoming established as playing an equally important role as classical protein–protein associations in promoting biologically relevant binding events in the cell, there is great interest in structurally mapping out linear motif-binding protein surfaces. The Eukary­otic Linear Motif database contains thousands of occurrences of LMs in various organisms (Gould *et al.*, 2010 ▶) and it is estimated that the human proteome may contain more than 10 000 LMs (Petsalaki & Russell, 2008 ▶). The moderate binding affinity of LM-containing peptides can hinder successful crystallization of the desired protein–peptide complex. Because linear motif-containing peptides are often unstructured alone and the energy gained upon crystal packing between symmetry mates may be on a par with the binding energy of the complex, the *bona fide* peptide-binding protein surface may mediate crystal packing rather than physiological LM binding.

Mitogen-activated protein kinase (MAPK)-binding linear motifs bind to the MAPK docking groove and represent a functionally well characterized linear binding-motif class (Garai *et al.*, 2012 ▶). Crystallization of docking-motif (D-motif) peptides with MAPKs may serve as a paradigm for the challenges of protein–peptide crystallization in general (Chang *et al.*, 2002 ▶; Heo *et al.*, 2004 ▶; Zhou *et al.*, 2006 ▶; Liu *et al.*, 2006 ▶; ter Haar *et al.*, 2007 ▶; Ma *et al.*, 2010 ▶; Garai *et al.*, 2012 ▶). These short peptides are 7–17 amino acids in length; they are unstructured without their binding partners and they bind to their cognate MAPKs with binding affinities of 1–10 µ*M* (Garai *et al.*, 2012 ▶). Previously, we attempted the crystallization of three different MAPKs (ERK2, p38α and JNK1) with different linear D-motif-containing peptides to gain structural insight into their MAPK binding specificity (Garai *et al.*, 2012 ▶). In this study, we describe our experiences in the crystallization of ERK2–docking peptide complexes. We set out to crystallize the wild-type protein (ERK2 WT) with six different peptides (Garai *et al.*, 2012 ▶). Unfortunately, ERK2 readily crystallized in the apo form and we only managed to grow protein–peptide cocrystals with the docking peptide from MNK1 (pepMNK1), which is an ERK2 substrate. Analysis of crystal-packing contacts subsequently revealed that the protein–peptide binding surface of ERK2 WT was blocked by a symmetry-related kinase molecule in all apo crystals, while ERK2–pepMNK1 crystals could ‘luckily’ form because this peptide mediated a different type of crystal packing. The peptide was engaged in crystal contacts with an ERK2 symmetry molecule (Supplementary Fig. S1^1^). Other peptides, however, could not mediate this unique crystal packing as they differed in length and sequence. Therefore, we decided to devise a strategy by which the trial-and-error nature of ERK2–docking peptide cocrystallization projects could be rationally alleviated.

## 2. Experimental procedures

ERK2 (UniProt ID P28482) was expressed in *E. coli* with an N-­terminal histidine tag, which was subsequently removed using TEV protease. The protease cleavage leaves a glycine–serine dipeptide N-­terminal to the first ERK2 residue. The expression and purification of ERK2 is described in further detail in Garai *et al.* (2012 ▶). Briefly, recombinant ERK2 was subjected to affinity purification on Ni-Sepharose, cleaved using TEV protease and loaded onto a RESOURCE Q ion-exchange column. The protein was eluted using an NaCl gradient, concentrated to 10 mg ml^−1^ and stored in buffer (20 m*M* Tris, 50 m*M* NaCl, 10% glycerol, 2 m*M* β-mercaptoethanol pH 8) at 193 K. ERK2 surface mutations were introduced by the QuikChange site-directed mutagenesis protocol and mutant proteins were expressed and purified in the same way as wild-type ERK2.

All crystallization experiments were performed in standard sitting-drop vapour-diffusion setups at 296 K. ERK2 (150–200 µ*M*) was crystallized using a twofold molar excess of chemically synthesized docking peptides in the presence of 2 m*M* AMPPNP and MgCl_2_. All peptides were synthesized on an ABI 431A peptide synthesizer using the Fmoc strategy.

For each ERK2–docking peptide complex we used an in-house 96-­condition PEG-based grid screen. This screen consisted of only low ionic strength (less than 200 m*M* salt) conditions, in which the pH and molecular weight of the PEGs were systematically varied from pH 5.5 to 8.5 and from PEG 200 to PEG 20 000, respectively. In addition, we also used low ionic strength commercial sparse-matrix screens (The PEGs and PEGs II Suites from Qiagen) for the initial crystallization trials of all complexes.

All crystals were flash-cooled after adding ∼15% glycerol to the mother liquor as a cryoprotectant. Crystals were tested on a Rigaku R200 rotating-anode X-ray generator at the Institute of Chemistry, Eötvös Loránd University and diffraction data sets were collected on the PXI or PXIII beamlines of the Swiss Light Source, Villigen, Switzerland. All data were processed with *XDS* (Kabsch, 2010 ▶). The phase problem was solved by molecular replacement with *Phaser* (McCoy *et al.*, 2007 ▶) using PDB entry 2gph (Zhou *et al.*, 2006 ▶) as a starting model. Structure refinement was carried out using *PHENIX* (Adams *et al.*, 2010 ▶) and structure remodelling and building was performed in *Coot* (Emsley *et al.*, 2010 ▶) (Table 1 ▶).

## 3. Results

**Figure 1:**
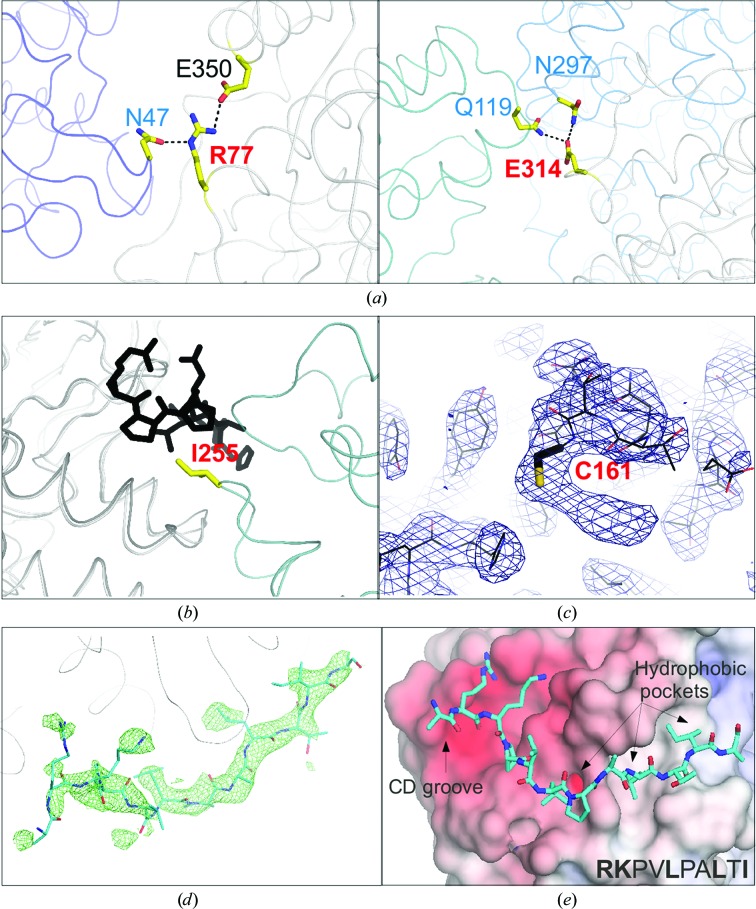
Surface engineering of ERK2 to interfere with ‘undesired’ crystal packing. (*a*) Arg77 forms hydrogen bonds with Asn47 from a symmetry mate in the crystal (left panel) and Glu314 interacts with Gln119 and with Asn297 from two ERK2 WT molecules (right panel). (*b*) The side chain of Ile255 from an ERK2 symmetry mate (coloured teal) occupies the hydrophobic groove in the apo ERK2_AA structures. The superimposed ERK–pepDCC complex structure (PDB entry 3o71; Ma *et al.*, 2010 ▶), shown in dark grey (MAPK) and black (pepDCC), on ERK2_AA demonstrates that this type of crystal packing is incompatible with D-motif peptide binding. (*c*) The 2*F*_o_ − *F*_c_ electron-density map contoured at 1σ for the final apo ERK2_AAG structure shows strong and continuous density for the side chain of Cys161. This indicates adduct formation with β-mercaptoethanol at this cysteine residue. (*d*) *F*_o_ − *F*_c_ simulated-annealing OMIT map contoured at 2σ for the ERK2–pepMKK2 complex. (*e*) Crystal structure of the ERK2–pepMKK2 complex. The ERK2 surface is coloured according to its electrostatic potential (red, negative; blue, positive).

MKK2 is one of the upstream activator kinases of ERK2 that binds its MAPK substrate with a linear docking motif (Garai *et al.*, 2012 ▶). In order to explore the structural basis of this interaction, we attempted to crystallize ERK2 with a docking peptide from MKK2 (pepMKK2). Single crystals grew in 20–25%(*w*/*v*) PEG 6000 buffered with 0.1 *M* MIB (a composite buffer comprised of malonate, imidazole and boric acid) pH 6.5. However, structure solution subsequently revealed that these crystals did not contain the chemically synthesized peptide: the MAPK docking groove was occupied by a crystallographic symmetry-related kinase molecule. In order to devise a strategy to prevent this kind of crystal packing in which the docking groove is blocked by a symmetry mate, we first analyzed the packing interactions of apo ERK2 and the ERK2–pepMNK1 protein–peptide complex using *PISA* (Krissinel & Henrick, 2007 ▶). Comparison of the main crystal contacts observed in these two ERK2 structures revealed that Arg77 and Glu317 make two prominent hydrogen-bond-mediated contacts with symmetry mates in the apo form, while these are not engaged in the packing of the ERK2–pepMNK1 complex (Fig. 1 ▶ *a*). Therefore, we replaced these residues by alanines (ERK2_AA) to ‘weaken’ the contacts in the crystal with a symmetry mate occupying the MAPK docking groove.

Crystals grew readily in 20–25%(*w*/*v*) PEG 6000 buffered with 0.1 *M* MIB pH 5.5 using this new ERK2 construct in the presence of pepMKK2, but structure solution subsequently showed that these crystals were of apo ERK2_AA (Table 2 ▶). We noticed that in both types of apo structure (ERK2 WT and ERK2_AA) the side chain of Ile255 of a symmetry-related MAPK molecule occupied one of the important linear motif-binding hydrophobic pockets of the MAPK docking groove (Fig. 1 ▶ *b*). We decided to directly mutate the contact residue (Ile255) to glycine (ERK2_AAG) to make this type of crystal packing less probable. Using the ERK2_AAG construct, we could grow small crystals in 25–30%(*w*/*v*) PEG 3000 buffered with 0.1 *M* Tris pH 8.5. The size of these crystals was then increased by macroseeding. Unfortunately, these crystals contained apo ERK2_AAG; however, the space group and packing were different compared with previous apo ERK2 structures. Although the packing of the apo ERK2_AAG crystals was compatible with peptide binding because the docking groove was ‘open’, Cys161 in the MAPK docking groove made a (2-hydroxyethyl)­thiocysteine adduct with β-mercapto­ethanol that was added to avoid oxidation during macroseeding (Fig. 1 ▶ *c*). This modification of Cys161 was likely to occur during the longer time period required for macroseeding crystallization experiments and interfered with docking-peptide binding. Oxidation of the corresponding residue (Cys162) during the crystallization of p38α MAPK has been observed previously (Patel *et al.*, 2004 ▶). We introduced a cysteine-to-serine mutation into the ERK2-AAG construct (ERK2_AAGS) and used this for crystallization. The ERK2_AAGS-pepMKK2 crystals finally contained the peptide and diffracted to 2.2 Å resolution (Figs. 1 ▶ *d* and 1 ▶ *e*).

Parallel to our trials with pepMKK2, we attempted to crystallize ERK2 with two other peptides (pepRSK1 and pepRSK1_SQAA). PepRSK1 contains a reverse D-­motif from a downstream MAP kinase-activated protein kinase (MAPKAP) that is a known ERK2 substrate (RSK1; Garai *et al.*, 2012 ▶). PepRSK1_SQAA is a mutated version of pepRSK1 in which intra-peptide hydrogen-bond stapling interactions were removed by replacing a serine and a glutamine residue by alanines (Garai *et al.*, 2012 ▶). In order to grow complex crystals with RSK1 peptides, we used the same sparse-matrix screens that were also used for pepMNK1 and pepMKK2. However, apo ERK2 crystals were always obtained when wild-type ERK2 was used. Finally, we obtained complex crystals without the need for macroseeeding by using the ERK2_AA construct in the presence of pepRSK1 or pepRSK1_SQAA [in 25–30%(*w*/*v*) PEG 6000 buffered with 0.1 *M* MES pH 6.5; Table 2 ▶ and Supplementary Fig. S2].

## 4. Discussion

In summary, we were successful in interfering with the prevalent crystal packing of ERK2 observed in many apo structures. Our goal was to disfavour crystal packing in which the ERK2 peptide-binding surface is blocked by a symmetry molecule (‘closed’) and to promote new crystal packing in which the peptide-binding surface is ‘open’. Surface mutations allowed ERK2 to crystallize with diverse packings and some of them were compatible with docking-peptide binding. These new packing arrangements were indeed more ‘relaxed’, as expected, and this seemed to increase the chance of growing protein–peptide cocrystals. In addition, the bound peptides (*e.g.* pepMKK2, pepRSK1 and pepRSK1_QAA) were not involved in crystal packing in the new cocrystals. For flexible linear motifs this is more favourable in order to capture them in their physiologically relevant binding geometry.

Currently, examples of crystal engineering to increase the crystallizability of protein constructs or to make poor-quality crystals diffract better are more abundant in the literature compared with examples involving negative selection against ‘unwanted’ crystal-packing interactions (Heinz & Matthews, 1993 ▶; Lawson *et al.*, 1991 ▶; Yamada *et al.*, 2007 ▶; Honjo *et al.*, 2008 ▶). It was found for aspartyl-tRNA synthetase that disruption of lattice contacts hinders crystallization and that the addition of contacts favours it (Charron *et al.*, 2002 ▶). However, removing lattice contacts may produce crystals with better diffraction resolution limits or may resolve twinning problems (Green *et al.*, 2001 ▶; Shimamura *et al.*, 2009 ▶). There are several methods of increasing the chance of crystallization for proteins if sparse-matrix screens fail (Derewenda, 2010 ▶). These involve changing the length of the protein construct or introducing chemical modifications on surface residues (Dale *et al.*, 2003 ▶; Walter *et al.*, 2006 ▶). For example, Zhou *et al.* (2006 ▶) covalently attached a docking peptide to ERK2 by concurrently introducing cysteines into the peptide and ERK2. An artificial disulfide bridge formed during crystallization that ensured docking-peptide binding; however, this artificial covalent bridge distorted the geometry of peptide binding in the MAPK docking groove. These trial-and-error strategies may be contrasted with a more rational approach: the surface-entropy reduction (SER) method, which is based on replacing small clusters of two to three surface residues characterized by high conformational entropy with alanines (Derewenda & Vekilov, 2006 ▶). Furthermore, disruption of known common crystal contacts may also be part of complex crystal-engineering efforts, as reported for HIV-1 reverse transcriptase and diphthine synthase (Bauman *et al.*, 2008 ▶; Mizutani *et al.*, 2008 ▶).

‘Unwanted’ crystal contacts may also hamper the crystallization of larger protein–protein complexes. Selmer *et al.* (2012 ▶) had difficulties in crystallizing the 70S ribosome in complex with the EF-G translational factor because the ribosomal L9 protein from a symmetry mate in the crystal blocked the area responsible for EF-G binding. The problem was solved by expressing and crystallizing ribosomes without the L9 ribosomal protein; these readily crystallized in complex with different translational factors. It is acknowledged that the idea of a surface-engineering-based approach involving negative selection against specific lattice contacts is not new (Charron *et al.*, 2002 ▶; Green *et al.*, 2001 ▶; Shimamura *et al.*, 2009 ▶); however, we are not aware of other studies in which this has been exploited for difficult protein–peptide crystallization problems. We believe that a more widespread application of similar rational approaches to those described for ERK2 in this study could be a great asset in tackling other difficult protein–peptide complex crystallization projects, particularly where the protein–peptide interface shows a propensity for mediating ‘unwanted’ crystal packing.

## Supplementary Material

PDB reference: ERK2_AA–pepRSK1_SQAA, 4h3p

PDB reference: ERK2_AAGS–pepMKK2, 4h3q

Supplementary material file. DOI: 10.1107/S0907444912051062/bw5412sup1.pdf

## Figures and Tables

**Table 1:** Crystallographic data-collection and refinement statistics for ERK2–docking peptide complexes Values in parentheses are for the highest resolution shell.

	ERK2_AA–pepRSK1_SQAA	ERK2_AAGS–pepMKK2
Data collection		
Space group	*P*1	*P*2_1_2_1_2_1_
Unit-cell parameters (Å, °)	*a* = 41.5, *b* = 58.8, *c* = 79.2, α = 100.9, β = 99.0, γ = 90.0	*a* = 41.8, *b* = 58.5, *c* = 159.2, α = β = γ = 90.0
Resolution (Å)	42.37–2.3 (2.382–2.300)	47.15–2.2 (2.279–2.200)
*R*_merge_ †	0.048 (0.322)	0.056 (0.611)
〈*I*/σ(*I*)〉	11.15 (2.43)	14.14 (2.25)
Completeness (%)	94.95 (93.31)	99.03 (96.49)
Multiplicity	1.8 (1.8)	3.3 (3.3)
Refinement		
No. of unique reflections	30568 (2987)	20375 (1922)
*R*_work_/*R*_free_ ‡	0.178/0.223	0.181/0.231
No. of atoms		
Macromolecules	5687	2902
Ligands	62	31
Waters	177	84
Average *B* factors (Å^2^)		
Wilson *B* factor	35.1	41.7
Macromolecules	45.0	59.5
Solvent	38.8	44.2
R.m.s. deviations from ideal values		
Bond lengths (Å)	0.009	0.010
Bond angles (°)	1.35	1.41
Ramachandran analysis§, residues in (%)		
Favoured regions	87.5	86.6
Allowed regions	12.2	13.1
Disallowed regions	0.3	0.3
PDB code	4h3p	4h3q

† *R*_merge_ = 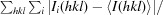 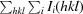.

‡ *R*_work_ = 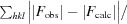 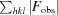, where *F*_obs_ and *F*_calc_ are the observed and calculated native structure factors, repectively. *R*_free_ is the same as *R*_work_ but calculated using 5% of the total reflections which were chosen randomly and omitted from the refinement.

§ Ramachandran analysis was carried out using *PROCHECK* (Laskowski *et al.*, 1993 ▶).

**Table 2:** Different apo ERK2 crystals and ERK2–docking peptide complexes The binding affinity of peptides to ERK2 are from Garai *et al.* (2012 ▶); amino acids in consensus sequence positions are shown in bold.

		Unit-cell parameters									
Construct	Peptide	*a* (Å)	*b* (Å)	*c* (Å)	α (°)	β (°)	γ (°)	Space group	Resolution (Å)	Peptide sequence	Binding affinity (µ*M*)
ERK2 WT	Apo	44.9	65.3	116.7	90.0	90.0	90.0	*P*2_1_2_1_2_1_	1.55	—	—
ERK2_AA	Apo	44.7	71.5	121.1	90.0	90.0	90.0	*P*2_1_2_1_2_1_	1.90	—	—
ERK2_AAG	Apo	86.5	86.5	311.1	90.0	90.0	120.0	*H*32	2.50	—	—
ERK2 WT†	MNK1	65.4	65.9	95.0	90.0	90.0	90.0	*P*2_1_2_1_2_1_	1.55	**M**K**L**SP**P**SKSR**L**AQ**RR**ALA	0.7
ERK2_AA†	RSK1	41.7	59.0	155.7	90.0	90.0	90.0	*P*2_1_2_1_2_1_	2.40	**P**Q**L**KP**I**ESSI**L**AQ**RR**VRKLSPTTL	0.3
ERK2_AA	RSK1_SQAA	41.5	58.8	79.2	100.9	90.0	90.0	*P*1	2.30	**P**Q**L**KP**I**EASI**L**AA **RR**VRKLSPTTL	2
ERK2_AAGS	MKK2	41.8	58.5	159.2	90.0	90.0	90.0	*P*2_1_2_1_2_1_	2.20	R**RK**PV**L**PA**L**T**I**NP	8

† These ERK2–docking peptide complexes were reported in Garai *et al.* (2012 ▶).

## Footnotes

1 Supplementary material has been deposited in the IUCr electronic archive (Reference: BW5412). Services for accessing this material are described at the back of the journal.
