# Supplementary material for: Protein–peptide complex crystallization: a case study on the ERK2 mitogen-activated protein kinase
Source: Acta Crystallogr D Biol Crystallogr. 2013 Feb 16;69(Pt 3):486–9. doi: 10.1107/S0907444912051062 (PMC3605046; doi:10.1107/S0907444912051062)
Supplement: Supplementary file 1 [file d-69-00486-sup1.pdf]

Supplementary Material

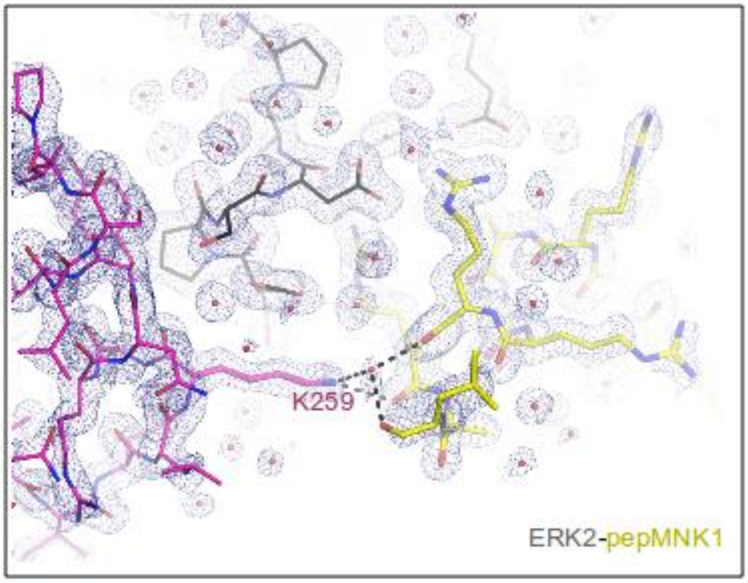

Figure S1. Peptide mediated crystal packing interactions in the ERK2-pepMNK1 complex.

Lys259 from an ERK2 symmetry mate (in magenta) forms crystal packing interactions (direct and water mediated H-bonds) to pepMNK1 (in yellow) bound to ERK2 (in gray). (2*F*o-*F*c map with the final ERK2 WT-pepMNK1 model contoured at 1σ. PDB ID: 2Y9Q; Garai et al., 2012)

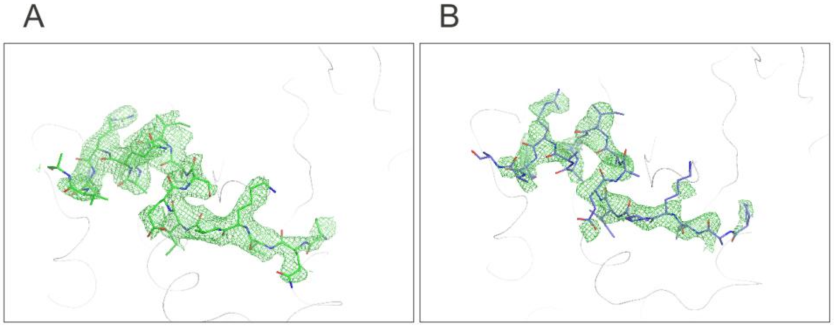

Figure S2. Electron density maps for MAPK-docking peptides complexes.

(A) F_o-F_c simulated annealing omit maps contoured at 2σ for the ERK2_AA-pepRSK1 complex.

(B) F_o-F_c simulated annealing omit maps contoured at 2σ for the ERK2_AA-pepRSK1_SQAA complex.
